# Contribution of Janus-Kinase/Signal Transduction Activator of Transcription Pathway in the Pathogenesis of Vasculitis: A Possible Treatment Target in the Upcoming Future

**DOI:** 10.3389/fphar.2021.635663

**Published:** 2021-03-29

**Authors:** Roberto Bursi, Giacomo Cafaro, Carlo Perricone, Ilenia Riccucci, Santina Calvacchi, Roberto Gerli, Elena Bartoloni

**Affiliations:** Rheumatology Unit, Department of Medicine and Surgery, University of Perugia, Perugia, Italy

**Keywords:** JAK/STAT, vasculitis, giant cell arteritis, Takayasu arteritis, Behçet’s disease, JAK inhibitors

## Abstract

Janus-kinase (JAK) and signal transduction activator of transcription (STAT) signal transduction pathway is involved in a wide range of physiological and pathological processes, including in the pathogenesis of several autoimmune diseases. Data supporting the role of JAK/STAT in the development of vasculitis are limited and mostly focused on large vessel vasculitis and Behçet’s disease. In this review, we provide a thorough picture of currently available evidence on the topic, gathered from *in vitro* experiments, animal models and human real-life data, analyzing the rationale for the use of JAK inhibitors for the management of vasculitis. Overall, despite a very strong biological and pathogenic basis, data are too few to recommend this therapeutic approach, beyond very severe and refractory forms of vasculitis. However, for the same reasons, a strong scientific effort in this direction is indeed worthwhile.

## Introduction

Vasculitides are a heterogeneous group of systemic inflammatory diseases characterized by the inflammation of the wall of blood vessels. The etiology is mostly unknown and pathogenesis still only partially understood. They are usually classified on the basis of the vessel dimension into large, medium and small vessel vasculitides, but they also differ in terms of epidemiology, clinical picture, prognosis and treatment (Watts and Robson, 2018).

Janus-kinase (JAK) and signal transduction activator of transcription (STAT) are the main players of a transduction pathway named JAK/STAT, involved in a wide range of physiological and pathological processes. JAKs are phosphotranspherases, i.e., enzymes able to transfer a phosphate residue from adenosine-tri-phosphate (ATP) to another substrate. JAKs do not have any receptorial function and are bound to various cytokine receptors. Upon ligand binding to their membrane receptors, JAKs are activated, and phosphorylate STATs to form a phosphorylated (*p*)-STAT dimer that is capable of migrating into the nucleus and inducing DNA transcription.

Four JAKs and seven STATs have currently been identified, named JAK1, JAK2, JAK3, tyrosine kinase (TYK)2 and STAT1, STAT2, STAT3, STAT4, STAT5A, STAT5B, STAT6, respectively. The specificity of signal transduction is determined by the numerous combinations among the isoforms of the three main players of JAK/STAT pathway, the receptor, JAK and STAT.

JAK/STAT is only one of the several pathways involved in intracellular signal transduction. In fact, some of the most well known cytokines involved in the pathogenesis of autoimmune and inflammatory diseases do not signal through JAKs, including tumor necrosis factor (TNF)α and interleukin (IL)-17. However, STATs are indeed involved in their transcription modulation.

On the contrary, all type I and type II cytokine receptors signal through JAK/STAT pathway. It is well beyond the purpose of this review to list and analyze the role of all these mediators. However, in order to provide a general but clear idea of how extensive the activity of JAK/STAT pathway is, it is necessary to mention the most well known pro- and anti-inflammatory mediators (IL-2, IL-6, IL-21, IL-12, IL-35, interferon—IFN-α, IFN-γ, IL-22, IL-10) and growth factors, such as erythropoietin, granulocyte-colony stimulating factor, granulocyte-monocyte-colony stimulating factor, that signal through it.

It is therefore quite intuitive how important this pathway is for hemopoiesis and host defense. Unsurprisingly, loss-of-function mutations of JAKs and STATs have been associated to a wide spectrum of immunodeficiencies, ranging from severe combined immunodeficiency (SCID) to milder forms of impaired immune responses. On the contrary, gain-of-function mutations have been associated with the development of autoimmunity (rheumatoid arthritis–RA, systemic lupus erythematosus–SLE, etc.) and hematologic malignancies.

Presently, commercially available JAK inhibitors have been approved for the treatment of RA, psoriatic arthritis (PsA) and ulcerative colitis (UC), with numerous preliminary data showing a potential good efficacy in other autoimmune conditions, including SLE.

One of the main features differentiating the various JAK inhibitors is their selectivity for the JAK subtypes. Currently available molecules are not selective for one specific isoform, though show a variable grade of affinity with a preferential binding to one or more subtypes. This aspect may represent an advantage, because, by inhibiting multiple isoforms, more cytokine signals are blocked. However, lack of selectivity is one of the main causes of the most frequent adverse reactions, such as cytopenia as a consequence of the inhibition of the JAK2 isoform. Numerous selective JAK inhibitors are currently under investigation, although it is still not clear whether they will provide a real advantage for the management of inflammatory diseases (Schwartz et al., 2016).

To our knowledge, this is the first published review presenting all currently available data on the potential benefit of the use of JAK inhibitors for the treatment of vasculitides. This area is still largely unexplored. However, in consideration of the great burden severe and unresponsive forms of vasculitis represent in terms of survival and quality of life, the exploration of innovative treatment strategies is of utmost importance.

## Janus-Kinase/Signal Transduction Activator of Transcription Pathway in the Pathogenesis of Vasculitis

**FIGURE 1:**
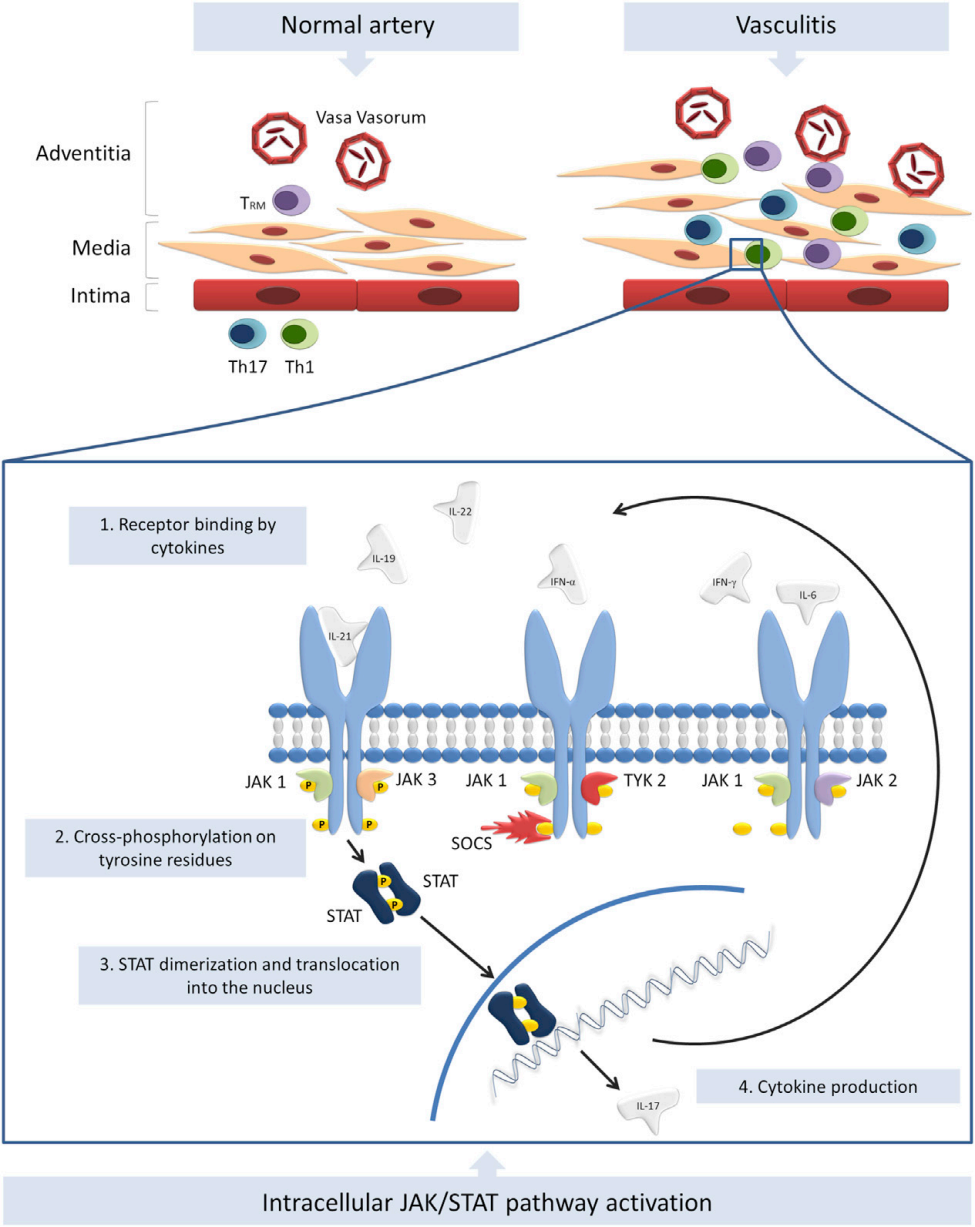
Summary of the current evidence on the role of JAK/STAT pathway in the pathogenesis of vasculitis. T_RM_, Th1 and Th17 cells infiltrate the vessel wall and induce inflammation. Numerous cytokines are secreted by T cells and innate immunity cells and activate JAK/STAT pathway. Other cytokines, such as IL-17 are therefore produced an secreted, further amplifying the inflammatory response. The modulation of SOCS transcription may be another altered mechanism involved in the pathogenesis of vasculitides.

As mentioned above, JAK/STAT pathway activation is tightly linked to the production of multiple cytokines, including type I and II IFN and thus they are involved in the pathogenesis of numerous autoimmune diseases, including vasculitis. Scientific data on the topic are very scarce and the few studies available are mostly focused on giant cell arteritis (GCA), Takayasu arteritis (TKA) and Behçet’s disease (BD). A graphical summary of the current evidence on the role of JAK/STAT pathway in the pathogenesis of vasculitis is provided in Figure 1.

GCA and TKA are the two most prevalent forms of large vessel vasculitis, preferentially involving the aorta and its larger branches. TKA is more common in young women, mostly in the second and third decade of life, while GCA typically affects people aged 50 years or older (Koster and Warrington, 2017; Watanabe et al., 2020). Differently, BD is a multisystemic chronic inflammatory disease, potentially involving small, medium and large vessels. It typically affects young adults aged 20–40 years and is more prevalent in countries along the ancient silk route.

Despite their etiology remains elusive, there is strong evidence supporting one or more antigens as the triggering factors at the basis of a dysregulated immune response and a consequent self-sustaining chronic inflammation in the context of a genetic predisposition (Carmona et al., 2015; Carmona et al., 2017; Hatemi et al., 2018; Zhang et al., 2018; Régnier et al., 2020).

Despite these forms of vasculitis share common features, such as vessel wall infiltration by immune cells, intimal hyperplasia, aneurysm formation, dissection and stenosis, the pathogenic process and cellular subsets involved are distinct. While T cells represent the key players in sustaining chronic inflammation in GCA and TKA, an aberrant response of the innate immune system is of utmost importance in the pathogenesis of BD (Dasgupta et al., 1989; Martinez-Taboada et al., 2001; Weyand and Goronzy, 2003; Weyand and Goronzy, 2013; Wen et al., 2016; Maleszewski et al., 2017a; Koster and Warrington, 2017; Puccetti et al., 2018; Zhang et al., 2018; Watanabe et al., 2020).

Due to its widespread biological functions, JAK/STAT pathway is potentially involved in all the pathogenic phenomena leading to the development of GCA, TKA and BD.

### Giant Cell and Takayasu Arteritis

As far as GCA and TKA are concerned, Th1 and Th17 cells represent the main players in the development of the diseases. It is well known that the former are strictly linked to STAT1, STAT2 and STAT4 activity, while the latter to STAT3. It is in fact possible to induce GCA on healthy human artery engrafted on SCID mice by reconstituting them with peripheral blood mononuclear cell (PBMCs) from GCA patients. As expected, pathological analysis of the grafts demonstrated a robust T cell infiltrate. Additionally, in arteritic tissue lesions from the same GCA mouse model, STAT1 and STAT2-dependent target genes are strongly upregulated and there is an increased production of cytokines like IFN-γ, IL-17 and IL-21 (Watanabe et al., 2017; Zhang et al., 2018).

Further supporting these data, a transcriptome analysis of CD4^+^ and CD8^+^ T cells performed on a very large cohort of patients with TKA, showed a significantly increased expression of numerous genes closely related to JAK/STAT pathways such as IL-12, IL-17, IL-19, IL-22 and type I and II IFNs. Additionally, a network analysis showed JAK/STAT pathway-involved genes among those with the highest number of connections, further supporting the hypothesis of their pivotal role in TKA (Weyand et al., 2012; Weyand and Goronzy, 2013; Maleszewski et al., 2017a; Koster and Warrington, 2017; Watanabe et al., 2017; Watanabe et al., 2020; Zhang et al., 2018; Régnier et al., 2020).

Beyond the articular involvement, collagen induce arthritis (CIA) rats, especially if given a high-fat-diet, develop an inflammatory infiltration of the aorta and can thus be employed as models of large vessel vasculitis. The expression of p-STAT3 in the aortic endothelium of CIA rats is significantly increased, suggesting a potential role of JAK/STAT activation in vascular inflammation (Cai et al., 2020).

Noteworthy, a new subtype of no-circulating T memory cells have been recently described in GCA arteritic lesions and named tissue-resident memory T cells (T_RM_), identified as CD69^+^ and CD103^+^. Unlike conventional T cells, T_RM_ do not migrate to secondary lymphatic stations and seem to locally exert a pro-inflammatory effect through the activation of JAK/STAT. Additionally, the presence of infiltrating active T_RM_ has been demonstrated in the vessel wall of grafted temporal arteries in the SCID chimera mice model of GCA. T_RM_ also appear to be implicated in driving long-lasting inflammation through the recruitment of CD4^+^ lymphocytes (Zhang et al., 2018).

Very few *in vitro* studies have investigated the biology of JAK/STAT activation in T cells from vasculitis patients. However, cultured CD4^+^ T cells from GCA patients, even in the absence of antigen-presenting cells, have been shown to spontaneously and preferentially differentiate towards a Th1 phenotype and to produce IFN-γ. JAK/STAT pathway proved essential for their survival and activation (Park and Kupper, 2015; Koster and Warrington, 2017; Zhang et al., 2018).

### Behçet’s Disease

As we described, the pathogenesis of BD is profoundly different from that of GCA and TKA, showing a far greater involvement of the innate immune system. However, T cells and JAK/STAT pathway still seem to play a key part in the development of the disease. In fact, the same pro-inflammatory cytokines (IL-12, IL-17, IL-23, IFN-γ), and T cell subsets (Th1 and Th17) have been shown to be increased in the peripheral blood and serum of BD patients compared to healthy controls (HC) (Puccetti et al., 2018). However, basal levels of STAT3 and p-STAT3 are significantly increased in CD14^+^ monocytes and CD4^+^ T cells of BD patients compared to HC. This aspect seems to be in contrast with the prevalent expression of STAT1-2 on STAT3, observed in GCA. Additionally, STAT3 polymorphisms associated to susceptibility to BD have been identified in a Chinese population (Hu et al., 2012; Tulunay et al., 2015; Abdi et al., 2018).

Despite the above-mentioned observations, the role of JAK/STAT pathway in BD is still not clear, with only few and partially conflicting data available in the literature. The great variability of the disease phenotype and its relatively low frequency in most countries probably represent the main reasons of such paucity of data and discrepancies. As an example of conflicting data, an Iranian study found that the gene of suppressor of cytokine signaling (SOCS) 1, a negative regulator of JAK/STAT pathway, was hypermethylated in BD patients compared to HC. This observation would confirm a reduced transcription of SOCS1 itself. However, other authors found that SOCS1 and SOCS3 expression may actually be increased in PBMCs and circulating neutrophils from BD patients (Hamedi et al., 2014; Abdi et al., 2018).

### Other Vasculitides

Data on other forms of vasculitis are very scarce and no direct evidence of an involvement of JAK/STAT pathway in their pathogenesis is available. However, some hints of a potential role of JAK/STAT may come from the evidence of an upregulation of STAT3 in the PB CD4^+^ and CD8^+^ T cells of polyarteritis nodosa (PAN) patients and of increased levels of CXCL10 (a STAT induced gene) in active Kawasaki disease (Rimar et al., 2016; Berthelot et al., 2020).

## Janus-Kinase Inhibition as a Strategy for the Treatment of Vasculitis

Similarly to the data regarding the pathogenic role of JAK/STAT pathway in vasculitis, the evidence of the effects of JAK inhibition is limited.

### *In Vitro* Studies

*In vitro* inhibition of JAK1/3 on PB-derived CD4^+^ T cells of GCA patients (which are known to spontaneously differentiate towards a Th1 phenotype) demonstrated a strong reduction of IFN-γ producing cells (Zhang et al., 2018). Similarly, the *in vitro* treatment of TKA patient-derived T cells with the JAK inhibitors ruxolitinib and tofacitinib showed a reduced expression of Th1 and Th17 cells, an increase of CD4^+^ T regulatory (Treg) cells and a reduction of CD25 expression by CD4^+^ and CD8^+^ cells. It has also been hypothesized that the inhibition of JAK1/3 may modulate macrophages and natural killer cells activity (Régnier et al., 2020; Watanabe et al., 2020) and reduce IL-17A-induced activation of human umbilical vein endothelial cells (HUVECs) through the inhibition of p-JAK1, p-JAK2, p-JAK3 and p-STAT3 (Cai et al., 2020).

### Animal Models

As briefly mentioned above, JAK/STAT signaling is essential to keep T_RM_ cells alive and functional in order to perpetuate chronic inflammation. Interestingly, despite long-term glucocorticoid treatment, the T cells infiltrating the arterial wall may persist and be responsible of perpetuating inflammation (Maleszewski et al., 2017b). By administering tofacitinib to an engrafted mouse model of GCA, the Authors showed a significant reduction of the T_RM_ population infiltrating the vessel wall, a disruption of their survival signals, a reduction in the expression of lineage-determining transcription factors (T-bet, RORC and BCL6). Consequently, the production of pro-inflammatory cytokines such as IFN-γ, IL-17 and IL-21 was reduced, thus demonstrating a key role of JAK/STAT pathway in this process (Zhang et al., 2018).

As far as the endothelium is concerned, JAK inhibition is able to significantly reduce the tissue levels of growth factors, such as platelet-derived growth factor, fibroblast growth factor 2, vascular endothelial growth factor and, consequently, inflammation-associated microangiogenesis and mio-intimal hyperplasia (Maleszewski et al., 2017a; Koster and Warrington, 2017; Zhang et al., 2018; Cid et al., 2020). Additionally, in CIA rats with aortitis JAK/STAT pathway inhibition is able to restore normal levels of p-STAT3 and to significantly decrease endothelial IL-17A expression, thus reducing the severity of vascular inflammation (Cai et al., 2020).

### Clinical Evidence

Real-life data on the efficacy of JAK inhibitors for the treatment of vasculitis are even fewer (mostly coming from case-reports of resistant TKA) and with contrasting results. Albeit one single case-report showed a treatment failure, most published cases demonstrate a significant improvement of refractory disease with a reduction of IL-6 and p-STAT5 serum levels, along with an increase of Treg/T effector cell ratio. Interestingly, tofacitinib appears to be particularly effective in TKA complicated by UC. This may be related to a different underlying genetic background of these subjects (Terao et al., 2015; Abdolahi et al., 2020; Kuwabara et al., 2020; Palermo et al., 2020; Régnier et al., 2020; Sato et al., 2020; Watanabe, 2020; Yamamura et al., 2020).

A possible explanation of the susceptibility of treatment-resistant vasculitis to JAK inhibition may be related to the ability of antigen presenting cells to stimulate Th1 and Th17 (both essential in the pathogenic process) through independent and distinct signals. Consequently, blocking a single cytokine may not be sufficient to control the disease (Weyand et al., 2012; Weyand and Goronzy, 2013; Cid et al., 2020). Similarly, in PAN different cytokines may stimulate JAK/STAT3 pathway, thus explaining the potential efficacy of JAK inhibitors where the blockade of IL-6 failed. In support of this, a case-report of a long-standing, refractory PAN patient describes a significant response to tofacitinib (Rimar et al., 2016).

Regarding the use of JAK/STAT inhibitors in BD, a pilot study conducted on refractory patients with very heterogeneous disease manifestations and severity showed an overall significant improvement, especially of cardiovascular and joint domains. However, a poor response on gastrointestinal involvement has been observed. Although a clear explanation of this phenomenon is still lacking, it may be important to consider the similarity of BD gastrointestinal involvement with Crohn’s disease, in which JAK inhibition is likely not effective (Abdi et al., 2018; Kuwabara et al., 2020; Liu et al., 2020; Sato et al., 2020).

Despite data are very limited, the potential benefit of JAK inhibitors for the treatment of vasculitis may have a significant impact on the management of patients, especially in case of treatment-resistance. For this reason, clinical trials aimed at evaluating the efficacy of JAK inhibitors in GCA (NCT03725202, NCT03026504) and TKA (NCT04161898, NCT04299971) are currently ongoing and preliminary data are expected soon.

## Discussion

In the last few years, the role of JAK/STAT pathway in the pathogenesis of several autoimmune diseases has acquired growing attention. Most of the data currently available are focused on RA, PsA and UC, for which the use of JAK inhibitors have been approved. However, due to the involvement of JAK/STAT in the vast majority of the inflammatory processes, it is reasonable to hypothesize a wider use of JAK inhibition, including for the treatment of vasculitis.

Very little data currently support the use of JAK inhibitors in clinical practice. However, the solid biological rationale and a few published case-reports may suggest considering this approach in very severe and refractory forms of vasculitis and represent a strong impulse to persevere on this scientific path.

Nonetheless, because JAK/STAT inhibition carries significant and potentially very severe complications, we are very far from being able to suggest a widespread use of JAK inhibitors in vasculitis.
